# Churchill regulates cell movement and mesoderm specification by repressing Nodal signaling

**DOI:** 10.1186/1471-213X-7-120

**Published:** 2007-11-02

**Authors:** Eric R Londin, Laura Mentzer, Howard I Sirotkin

**Affiliations:** 1 Department of Neurobiology and Behavior, Stony Brook University Stony Brook, New York, USA; 2 Graduate Program in Genetics, Stony Brook University Stony Brook, New York, USA

## Abstract

**Background:**

Cell movements are essential to the determination of cell fates during development. The zinc-finger transcription factor, Churchill (ChCh) has been proposed to regulate cell fate by regulating cell movements during gastrulation in the chick. However, the mechanism of action of ChCh is not understood.

**Results:**

We demonstrate that ChCh acts to repress the response to Nodal-related signals in zebrafish. When ChCh function is abrogated the expression of mesodermal markers is enhanced while ectodermal markers are expressed at decreased levels. In cell transplant assays, we observed that ChCh-deficient cells are more motile than wild-type cells. When placed in wild-type hosts, ChCh-deficient cells often leave the epiblast, migrate to the germ ring and are later found in mesodermal structures. We demonstrate that both movement of ChCh-compromised cells to the germ ring and acquisition of mesodermal character depend on the ability of the donor cells to respond to Nodal signals. Blocking Nodal signaling in the donor cells at the levels of Oep, Alk receptors or Fast1 inhibited migration to the germ ring and mesodermal fate change in the donor cells. We also detect additional unusual movements of transplanted ChCh-deficient cells which suggests that movement and acquisition of mesodermal character can be uncoupled. Finally, we demonstrate that ChCh is required to limit the transcriptional response to Nodal.

**Conclusion:**

These data establish a broad role for ChCh in regulating both cell movement and Nodal signaling during early zebrafish development. We show that *chch *is required to limit mesodermal gene expression, inhibit Nodal-dependant movement of presumptive ectodermal cells and repress the transcriptional response to Nodal signaling. These findings reveal a dynamic role for *chch *in regulating cell movement and fate during early development.

## Background

The establishment of the vertebrate body plan depends on a carefully orchestrated series of position-dependent cell interactions that determine the nature and proportion of cells that will populate each of the three germ layers. The movement of cells or their resistance to move, influences the inductive signals they will encounter. These signals initiate developmental programs that generate various differentiated cell types.

The series of dynamic cell movements during gastrulation positions cells to receive signals that will direct them to a given fate. In zebrafish, these movements include epiboly, internalization and convergence and extension movements. Epiboly is the process of spreading and thinning of the embryo during blastula and gastrula stages. Mesendodermal precursor cells are located at the margin in a thickened region termed the germ ring. These precursors are internalized resulting in the formation of an outer epiblast layer and inner hypoblast layer [1].

As the germ layers are specified, there is an antagonistic relationship between mesoderm and neural induction. Expansion of the mesoderm comes at the expense of the ectoderm; conversely, repression of mesoderm results in an expansion of the ectoderm [2-5]. FGF signaling has critical roles in specification and patterning of the mesoderm and neural ectoderm in mice, frogs, fish and the chick [6-14].

In many species, neural and mesoderm induction occur at similar times and in adjacent cell populations. How can FGF function in the seemingly contradictory roles as an inducer of mesoderm and neural tissue? One possibility is that different FGF effectors are present in the mesoderm and ectoderm to regulate its activity. One candidate FGF effector is the zinc finger transcription factor Churchill (*chch*) [15].

*chch *overexpression in *Xenopus *embryos results in suppression of the mesodermal marker *brachyury *[15]. Morpholino knockdown of *chch *in the chick epiblast results in inappropriate migration of epiblast cells through the primitive streak [15]. *chch *morpholino-injected cells emerged from the primitive streak and gave rise to paraxial mesoderm. This suggests that *chch *is required to limit ingression of the epiblast allowing those cells to become neural tissue. In addition, the chick experiments implicate Smad-interacting protein-1 (Sip1) as a direct target of *chch *and suggest that Sip1 is the major *chch *effector involved in blocking ingression of the epiblast [15].

Although the effect of *chch *in the assays in the frog and chick is the same (to limit mesoderm), the mechanisms of action in these two experiments likely differ. One difference is that cell movement is not thought to be required for mesoderm induction in the animal cap assay. The chick experiments do not address the question of whether the migration of *chch*-inhibited epiblast cells exposes them to mesoderm-inducing signals or whether they migrate because they have already acquired mesodermal properties. In order to elucidate the mechanisms of action of *chch*, we have undertaken a series of experiments to study the requirement for *chch *in the zebrafish and to address the roles of *chch *in cell migration and cell fate.

Here, we show that *chch *is required to limit mesodermal gene expression in zebrafish. During gastrulation, inhibition of *chch *results in an increase in transcript levels of mesodermal genes and a decrease in levels of ectodermal transcripts. In cell transplant experiments, cells with compromised *chch *activity are more motile than wild-type cells when transplanted to the epiblast of wild-type hosts. These cells leave the epiblast and migrate into the germ ring to acquire mesodermal cell fates. We found that both migration of *chch*-compromised donor cells and acquisition of mesodermal character depend on Nodal signaling. Finally, we demonstrate that *chch *is required to repress the transcriptional response to Nodal signaling. Together, these findings demonstrate that *chch *regulates cell fate by limiting the response to Nodal signals.

## Results

### chch inhibition produces axial and somite defects

**Figure 1:**
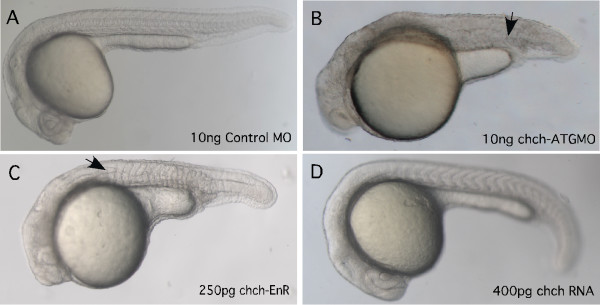
***chch *inhibition results in axial and somite defects**. The effect of inhibiting *chch *was examined using a morpholino and a dominant-negative construct. Microinjection of *chch*-ATGMO results in a broad, misshapen notochord (arrow) and misshapen somites and a shortened axis (B). Microinjection of *chch*-EnR, results in a similar phenotype as the morpholino, a broad, misshapen notochord, and enlarged and misshapen somites formed (C). *chch *mRNA overexpression produces embryos that are wild-type in appearance (D). Arrowheads point to the notochord. All embryos are 24 hpf.

Zebrafish *chch *sequence was previously reported [15] but the zebrafish *chch *has not been further characterized. We have determined that like in the chick, zebrafish *chch *is regulated by FGF signaling, but unlike in the chick expression is widespread and not limited to the prospective neural plate [16]. To examine the function of *chch *in zebrafish, we inhibited *chch *activity with a morpholino directed against the translation start site and a dominant-negative mRNA. Microinjection of *chch*-ATGMO produces embryos with enlarged and misshapen somites (Fig. 1B). In addition, these embryos have a short body axis and poorly formed anterior neural structures (Fig. 1B).

Since *chch *functions as a transcriptional activator [15], we generated a dominant-negative construct by fusing the zebrafish *chch *coding sequence to the *drosophila *engrailed repressor domain (*chch*-EnR). Microinjection of *chch*-EnR mRNA pronounced a similar phenotype to the morpholino; highly disorganized somites, a wide notochord and short axis were observed (Fig. 1C). Taken together, these results demonstrate that *chch *is essential for proper formation of the body axis and suggest that chch deficiency may result in convergence extension defects.

### chch is not required for expression of dorsal mesodermal markers

**Figure 2:**
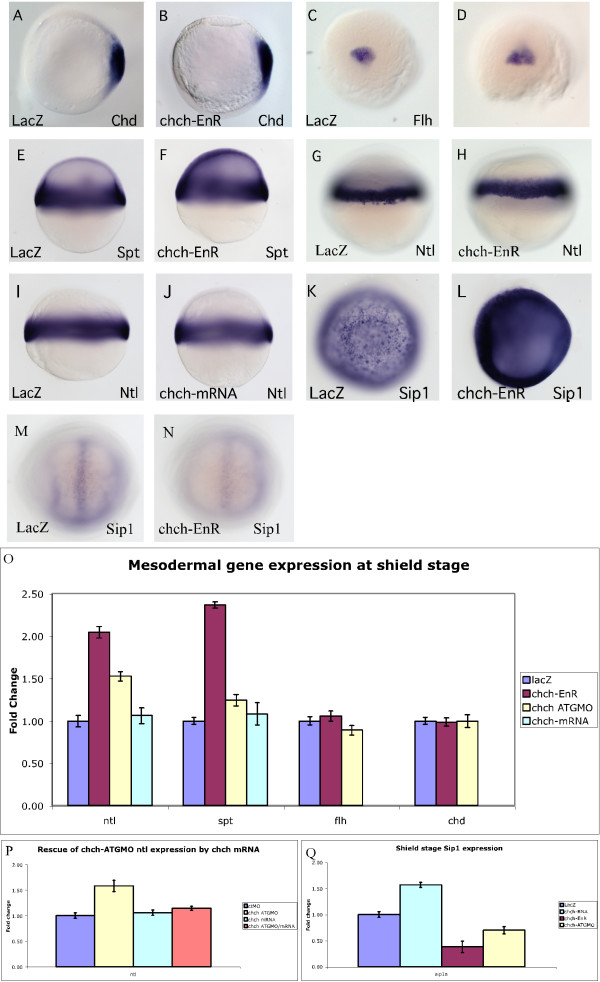
***chch *represses mesodermal markers**. Mesodermal markers were examined by RNA *in situ *hybridization and real-time PCR in embryos microinjected with *chch*-EnR mRNA. Inhibition of *chch *does not alter expression of dorsal mesodermal genes *chd *(A, B) or *flh *(C, D). Expression of mesodermal markers at shield stage, including *spt *(E-F) and *ntl *(G-H) are expanded slightly in *chch*-inhibited embryos. Overexpression of *chch *mRNA does not alter *no-tail *expression at shield stage (I-J). *chch *inhibition results in a decrease in Sip1 gene expression at bud stage (M-N), while *chch *activation results in Sip1 induction at shield stage (K-L). Real-time PCR analysis of mesodermal markers in *chch*-inhibited embryos (O). The fold change in transcript levels (y-axis) is graphed relative to control embryos following overexpression of *chch*-EnR mRNA, *chch*-ATGMO and *chch *mRNA. This analysis reveals that the domains of mesodermal genes are expanded at shield stage while dorsal mesodermal genes are unaffected. Conversely, overexpression of *chch *mRNA does not result in alteration of early mesodermal gene expression. The induction of *ntl *expression following *chch *inhibition with the ATGMO can be rescued by co-expression of *chch *mRNA (P). Real-time PCR analysis of Sip1 mRNA in *chch, chch*-EnR and *chch*-ATGMO treated embryos (Q). Induction of *chch *results in a 50% increase in Sip1 expression, conversely, inhibition of *chch *results in a 70% reduction of Sip1 expression. The fold change in Sip1 transcript levels (y-axis) is graphed relative to control embryos. Views: A-B, K-L are animal pole; C-D, M-N are dorsal; E-J are lateral.

To determine if the axial defects observed in *chch *compromised embryos stems from dorsal mesodermal defects, we examined dorsal mesodermal markers during early gastrulation by *in situ *hybridization and real-time PCR following microinjection of *chch*-EnR mRNA. At this stage, both chordin (*chd*) and floating head (*flh*) are expressed within the dorsal mesoderm. Following microinjection of *chch*-EnR mRNA, neither marker showed altered expression by RNA *in situ *hybridization (Fig. 2A–D) or real-time PCR (Fig. 2O). Other organizer-specific markers such as noggin and goosecoid were also examined (data not shown) and did not show a change in expression. Similarly, microinjection of *chch*-ATGMO did not produce changes in *chd *or *flh *expression by either *in situ *hybridization or real-time PCR (Fig. 2O and data not shown). These data suggests that repressing *chch *function does not alter initial specification of dorsal mesoderm but acts on subsequent steps in axial patterning or morphogenesis.

### chch regulates mesoderm specification

To determine if expression of other mesodermal genes is altered when *chch *function is reduced, we examined pan-mesodermal markers at the start of gastrulation. *In situ *hybridization and real-time PCR were used to assay expression of no tail (*ntl*), spadetail (*spt*) and *tbx6*. All of these markers showed significant increases in transcript levels at shield stage following microinjection of *chch*-EnR mRNA. When assayed by quantitative real-time PCR the mesodermal markers showed increases corresponding to as much as a to a 2–2.25-fold increase in transcript levels (Fig. 2O). The spatial alterations in gene expression were more subtle, as mesodermal markers showed slightly more robust expression (Fig. 2E–H and data not shown). Surprisingly, overexpression of *chch *mRNA did not alter mesodermal marker expression (Fig. 2I–J, O). Similarly, *chch *inhibition with the morpholino did not alter the spatial expression of mesodermal markers (data not shown) but slight increases in *ntl *and *spt *gene expression were observed by real-time PCR (1.5 and 1.25-fold increases respectively, Fig. 2O). Importantly, co-expression of *chch*-ATGMO along with *chch *mRNA that had been mutated to prevent morpholino binding rescued the increases in mesodermal gene expression seen following *chch *inhibition (Fig. 2P). Together, these data demonstrate that *chch *is required to limit mesodermal gene expression. However, unlike in *Xenopus *[15], ectopic *chch *is insufficient to repress *ntl*/*brachyury *expression.

Smad-interacting protein 1 (Sip1) is a direct target of *chch *in the chick [15] and a direct repressor of *Xenopus brachyury *[17]. Therefore, we asked whether the inability of ectopic zebrafish *chch *to repress *ntl *stemmed from a failure to induce Sip1 expression. We examined Sip1 expression by real-time PCR with primers generated from the Sip1 genomic locus (Ensembl gene Id: ENSDARG00000059564) and by RNA *in situ *hybridization with a cDNA probe generated with primers designed from that locus. Overexpression of *chch *mRNA results in large increases in Sip1 transcript levels when examined by RNA *in situ *hybridization (Fig 2L) and real-time PCR (Fig. 2Q). Conversely, inhibition of *chch *with either the dominant-negative construct (fig. 2N and 2Q) or the morpholino(Fig. 2Q) results in a decrease in Sip1 expression in the same assays. Together, these results reveal that zebrafish Sip1 is regulated by *chch*. Therefore, despite robust induction of Sip1, overexpression of zebrafish *chch *is not sufficient to alter *ntl *expression.

**Figure 3:**
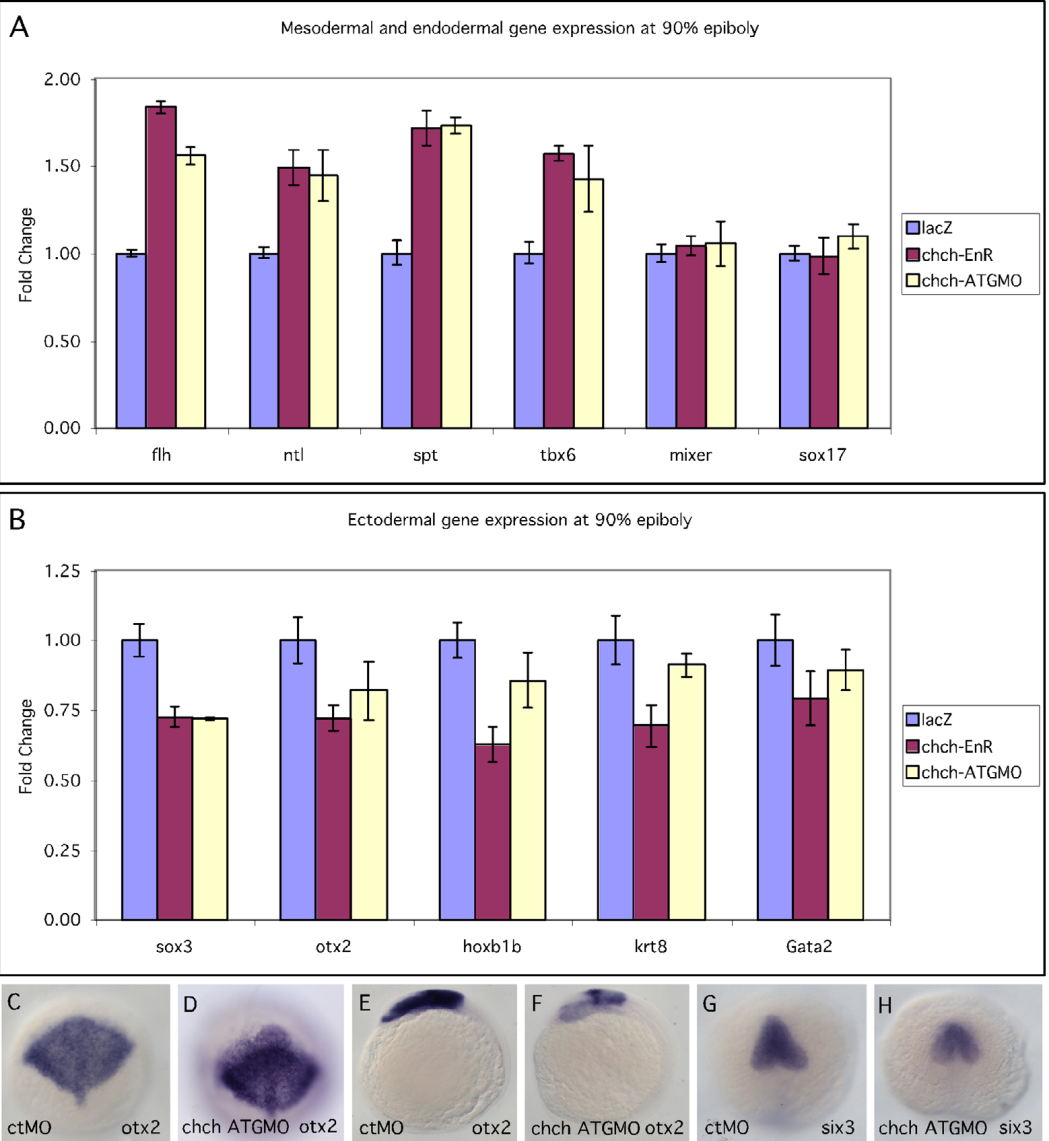
***chch *inhibition results in decreased ectodermal gene expression**. Real-time PCR analysis of mesodermal, ectodermal and endodermal markers during late gastrulation in *chch*-compromised embryos. The mesodermal markers *flh, ntl*, *spt*, and *tbx6 *(A), endodermal markers, *mixer *and *sox17b *(A) and ectodermal markers *otx2*, *hoxb1b*, *sox3*, *krt8 *and *gata2 *(B) were examined following microinjection of *chch*-EnR mRNA and *chch*-ATGMO. Mesodermal markers are increased, endodermal are unaffected while ectodermal marker levels are decreased. Expression of the neural genes *otx2 *at 90% epiboly (C-D) and 6-somites (E-F) and *Six3 *at 6-somites (G-H). C-D, G-H are dorsal views. E-F are lateral views.

We next assayed whether the increase in mesodermal gene expression following *chch *inhibition came at the expense of other germ layers. By late gastrulation, mesodermal markers in *chch*-inhibited embryos show a 50–75% increase compared to control embryos when assayed by real-time PCR (Fig. 3A). Conversely, the endodermal markers, *mixer *and *sox17*, do not show altered mRNA levels at late gastrulation following inhibition of *chch *activity (Fig. 3A). This contrasts ectodermal gene expression at late gastrulation. By RNA *in situ *hybridization, the expression domains of the neural gene *otx2 *were not obviously altered (Fig. 3C–D) by late-gastrulation. However, real-time PCR analysis revealed that mRNA levels of both neural and epidermal markers were consistently decreased in *chch *morphants and *chch*-EnR treated embryos. The anterior neural marker *otx2*, the posterior neural marker *hoxb1b*, the pan-neural marker *sox3 *and the epidermal markers *krt8 *and *gata2 *all showed decreased transcript levels (Fig. 3B). By early somitogenesis, the anterior neural markers *otx2 *and *six3 *both had reduced expression domains in *chch *morphants (Fig. 3E–H). Overexpression of *chch *mRNA did not result in a change in ectodermal or endodermal gene expression during late gastrulation (data not shown).

While we observed a slight spatial increase in expression of mesodermal markers at shield stage when *chch *function was abrogated, we were unable to detect a subsequent spatial decrease in ectodermal marker expression until early somite stages. However, the quantitative real-time PCR data demonstrates a consistent decrease in the levels of expression of ectodermal markers during gastrulation. The ectodermal deficits may be too subtle to be detected by *in situ *hybridization. These findings suggest that *chch *regulates mesodermal gene expression during early development.

### Inhibition of chch results in aberrant cell movements and cell fate changes

**Figure 4:**
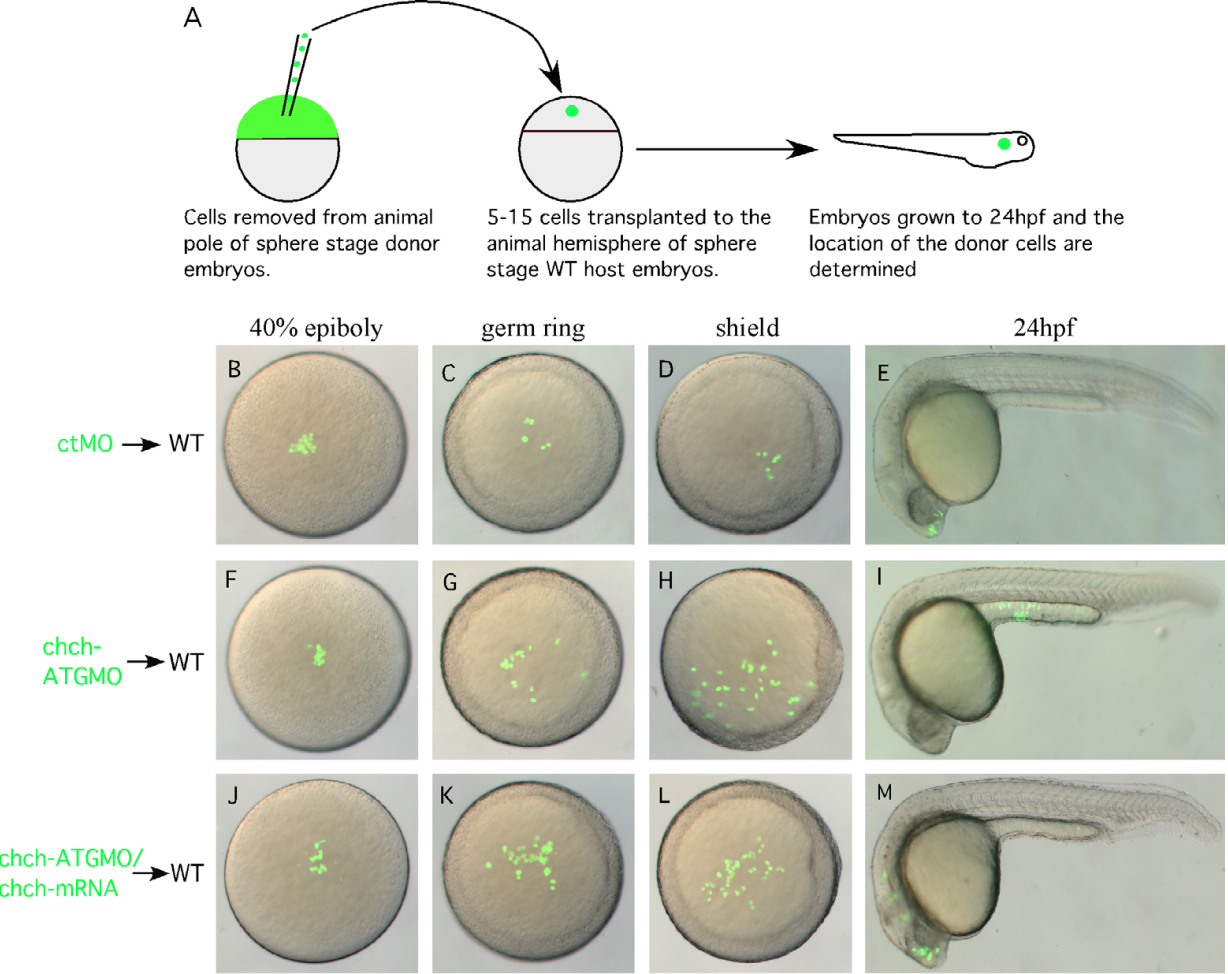
**chch inhibition results in inappropriate cell movements**. (A) A schematic representation of the transplantation scheme. ctMO or *chch*-ATGMO mRNA-injected donor cells were transplanted to the hemisphere of a sphere stage wild-type host. Embryos were photographed at 40% epiboly (B, F, J), germ ring (C, G, K) shield stage (D, H, L), and 24 hpf (E, I, M). The *chch *morphant cells (F-I) undergo greater spreading than the control cells and move toward the margin. At 24 hpf control cells are often found in anterior neural tissue (E) while the *chch *morphant cells are more frequently found in superficial cell layers of the trunk (I). The abnormal movement of *chch *morphant cells to superficial cell layers of the trunk is rescued by the injection of *chch*-mRNA (J-M).

We next examined whether repression of *chch *alters cell movements by tracking the behavior of *chch *compromised cells in a wild-type host. Sphere stage (mid-blastula) cells from ctMO or *chch*-ATGMO injected donor embryos were transplanted to the animal pole of similar stage wild-type hosts. Donor cell movements were observed at 40% epiboly, germ ring, shield stage and after 24 hpf (Fig. 4B–M). Control donor cells undergo limited movement and spreading (Fig. 4B–D). In contrast, *chch*-inhibited cells moved vegetally and spread much faster (Fig. 4F–H).

To determine whether the vegetal movement of *chch *inhibited cells resulted in a fate change, we recorded the position of the donor cells after 24 hpf. As expected, when transplanted to the animal pole, ctMO-donor cells generally became incorporated into anterior neural ectoderm (42/52 embryos 80.1%, Table 1, Fig. 4E). In a few embryos, ctMO-donor cells were found in the superficial cell layers in 3/42 embryos (7.1%, table 1) or in both neural tissue and superficial cell layers of the trunk 7/52 embryos (13.5%, Table 1). *chch *morphant donor cells behaved differently (Fig. 4I, Table 1). When transplanted to the animal pole of a wild-type host, 44/72 embryos (61.1%), *chch *morphant donor cells were observed in anterior neural structures. In the remaining embryos, the cells were spread over superficial layers of the trunk in 15/72 embryos (20.8%) or in both neural tissue and superficial cell layers of the trunk in 12/72 embryos (16.7%, Table 1). Importantly, the movement phenotype produced by the *chch*-ATGMO could be rescued by co-injection with *chch *mRNA (Fig. 4J–M). Unlike *chch *morphant donor cells which were restricted to the superficial layers of the trunk in 15/72 (20.8%) transplants, cells from *chch*-ATGMO + *chch *mRNA injected donors were never restricted to the superficial layers of the trunk (0/41 embryos, Fig 4J–M, Table 1). However, in 8/41 (19.5%) embryos, cells were observed in superficial layers of the trunk and in anterior neural tissue (Table 1), a rate comparable to the control transplant. Cells from *chch*-EnR mRNA injected donors also underwent similar spreading behavior as the *chch *morphant cells (data not shown).

**Table 1:** Transplantation of *chch *inhibited cells during mid-blastula stages results in inappropriate cell movements.

		**Location of cells at 24 hpf**			
		
Treatment	N	**Neural tissue**	**Superficial cells spread over the trunk**	**Neural and superficial cells spread over the trunk**	**Mesoderm**
CtMO	52-embryos	42-embryos	3-embryo	7-embryos	0-embryos
*chch*-ATG2MO	72-embryos	44-embryos	15-embryos	12-embryos	1-embryo
*chch*-ATG2MO/*chch mRNA*	41-embryos	33-embryos	0-embryos	8-embryos	0-embryos

**Figure 5:**
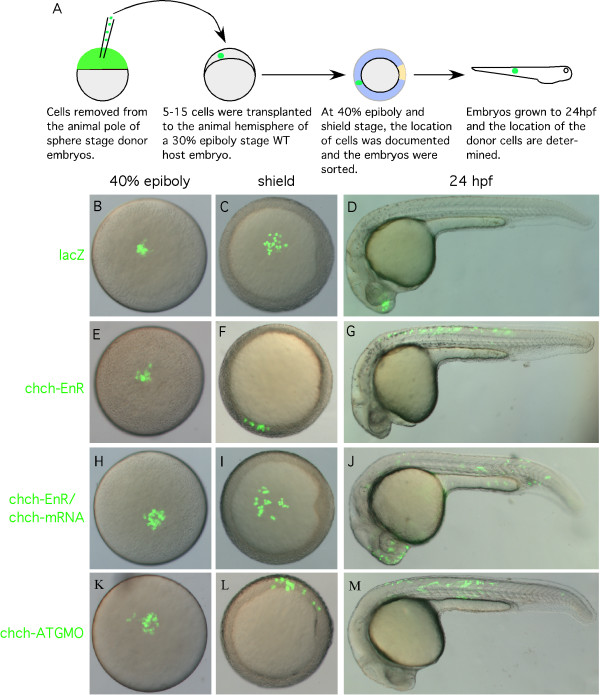
***chch*-inhibited cells leave the epiblast and enter the germ ring and become mesoderm**. (A) A schematic representation of the transplantation scheme. Cells from sphere stage LacZ, *chch*-EnR mRNA or *chch*-ATGMO injected donors were transplanted to the animal hemisphere of 30% epiboly (late blastula) wild-type embryos. Embryos were photographed at 40% epiboly (B, E, H, K), at the start of gastrulation (shield) (C, F, I, L) and after 24 hpf (D, G, J, M). As expected, LacZ cells remain within the ectoderm and take on ectodermal fates at 24 hpf (B-D). In contrast, *chch *inhibited cells often migrate from the presumptive ectoderm to the germ ring (E-G, K-M). These cells are often later found in the somites (G). The abnormal movement of *chch*-EnR cells into the germ ring is rescue by coinjection of *chch *mRNA (H-J). The images in each row are of the same embryo.

These results show that *chch *inhibition results in inappropriate cell movements. To determine if the increase in mesodermal gene expression observed in *chch*-inhibited embryos (Fig. 2) results from inappropriate cell movements, cell transplant experiments were performed to assay the behavior of *chch*-EnR and *chch*-ATGMO cells in wild-type hosts. Since transplantation of sphere stage (mid-blastula) *chch *compromised donor cells to sphere stage wild-type host resulted in movement of *chch *compromised donor cells to superficial cell layers, we performed a series of heterochronic transplants to determine if the vegetal migration could result in a mesodermal fate change. Here, sphere (mid-blastula) stage LacZ mRNA, *chch*-ATGMO or *chch*-EnR mRNA injected cells were transplanted to the animal hemisphere of 30% epiboly (late-blastula) stage embryos. Donor cell position was documented at 40% epiboly (5 hpf), shield stage (6 hpf) and 24 hpf (Fig. 5 and Table 2, 3).

**Table 2:** Transplantation of *chch*-inhibited cells undergo inappropriate movements during gastrulation

		**Location of Cells at shield**				
		
**Injected mRNA**	**N**^a^	**Dorsal Ectoderm**	**Ventral ectoderm**	**Shield**	**Lateral mesoderm**	**Ventral mesoderm**
LacZ	79	52	25	0	2	0
*chch*-EnR	110	38	28	10	20	14
*chch*-EnR/*chch*-mRNA	38	20	13	0	2	3
*chch*-ATGMO	51	26	17	1	5	2

^a^-Host embryos with cells in the presumptive ectoderm at 40% epiboly

**Table 3:** Transplantation of *chch*-inhibited cells undergo inappropriate movements during gastrulation and results in cells in the mesoderm.

			**Location of cells at 24 hpf**				
			
**Location of cells at shield stage**	**Injected mRNA**	**N**^b^	**Neural tissue**	**Epidermis**	**Notochord**	**Anterior somites**	**Posterior somites**
Shield	LacZ	0	0	0	0	0	0
	*chch*-EnR	10	2	0	6	2	0
	*chch*-EnR/*chch*-mRNA	0	0	0	0	0	0
	*chch*-ATGMO	1	0	0	1	0	0
Lateral Mesoderm	LacZ	1	0	0	0	1	0
	*chch*-EnR	20	2	0	4	12	2
	*chch*-EnR/*chch*-mRNA	2	0	0	0	2	0
	*chch*-ATGMO	5	0	0	0	2	3
Ventral Mesoderm	LacZ	0	0	0	0	0	0
	*chch*-EnR	14	1	1	0	10	2
	*chch*-EnR/*chch*-mRNA	3	0	0	0	2	1
	*chch*-ATGMO	2	0	0	0	1	1

^b^-Same embryos were scored at 40% epiboly, shield stage and 24 hrs

When LacZ cells were transplanted into the animal hemisphere of late-blastula embryos, donor cells remained within the ectoderm in 77/79 hosts (Fig. 5B–D, Table 3). Cells with compromised *chch *function behaved dramatically different in this assay (Fig. 5E–G). In 44/110 (40.0%) transplants, *chch*-EnR donor cells migrated from the epiblast to the germ ring by shield stage (Table 2). The effects of the *chch*-ATGMO were less pronounced but in 8/51 (15.7%) transplants, morphant cells migrated from the epiblast to the germ ring (Fig. 5K–M). Cells from donor embryos that had been co-injected with *chch*-EnR mRNA and *chch *mRNA tended to remain in the animal hemisphere (Fig. 5H–J, Table 2). In these transplants in only 5/38 (13.2%) hosts, was migration of donor cells to the germ ring observed. This demonstrates that the migration of *chch *deficient donor cells can be rescued with *chch *mRNA and indicates that the defect stems from knockdown of *chch *function.

To determine whether movement of *chch*-compromised cells to the germ ring correlated with a fate change in these cells, we determined their locations at 24 hpf. Of the *chch*-EnR donor cells that migrated to the germ ring, 38/44 were found in mesodermal structures at 24 hpf. Likewise, 8/8 *chch *morphant donor cells that migrated to the germ ring were later found in mesodermal structures. This demonstrates that *chch *compromised cells transplanted distant from the margin at 30% epiboly generated mesoderm while LacZ donor cells did not. In 6/44 transplants, *chch*-EnR donor cells migrated to the germ ring but did not give rise to mesoderm. This suggests that movement of these cells may not entirely be linked to acquisition of mesodermal character.

The location of the *chch *compromised donor cells after 24 hpf was often not characteristic of their position at shield stage. The *chch*-EnR cells that migrated out of the ectoderm and into the germ ring also acquired unexpected fates and were found in surprising locations. Cells located in the ventral germ ring would be expected give rise to posterior somites. Instead, these cells were found in more anterior somites (first 15 somites, 10/14 embryos) or were observed in the ectoderm (2/14 embryos) (Table 2). Cells located lateral to the shield would be expected to assume anterior somite fates. While a majority of these cells did give rise to anterior somites (12/20 embryos), cells were also located within the notochord (4/20 embryos) and neural tissue (2/20 embryos) (Table 3). Together, these results suggest that inhibiting *chch *results in several kinds of inappropriate cell movements including movement of presumptive ectodermal cells into the germ ring.

### Migration and fate change of chch compromised cells requires Nodal signaling

Our initial transplant experiments do not reveal whether the fate changes observed in *chch*-compromised donor cells result from a failure to limit cell movement or whether a fate change precedes the improper movement. Three models could account for the movement of *chch*-compromised donor cells to the germ ring and subsequent acquisition of mesodermal character that we observed: 1) *chch*-compromised donor cells may autonomously express mesodermal markers which drives both movement and acquisition of mesodermal character; 2) inappropriate movement of *chch*-compromised donor cells to the margin may result in exposure of donor cells to mesoderm inducing signals; or 3) *chch*-compromised donor cells may exhibit increased sensitivity to non-autonomous signals that drive migration and/or acquisition of mesodermal character. Since induction of trunk mesoderm requires Nodal signaling [3,18], we reasoned that blocking Nodal signaling after transplantation of *chch*-compromised cells would allow these models to be distinguished. If the first model is correct, migration of *chch *compromised cells and fate change will occur regardless of the state of Nodal signaling. If inappropriate movement allows *chch *compromised donor cells to come under the influence of the high levels of Nodal ligands present in the germ ring (model 2), we would observe migration of donor cells, but these cells would be unable to respond to mesoderm inducing signals. If the third model is correct, both migration and acquisition of mesodermal character will be blocked in *chch*-compromised donor cells when Nodal signaling is abrogated.

To test these hypotheses, cell transplantation experiments were performed with *chch*-EnR donor cells placed into the animal hemisphere of wild-type hosts. We used *chch*-EnR donors instead of *chch *morphant donors because these cells had a greater tendency to migrate from the epiblast to the germ ring (Table 2). Both donors and hosts were maintained in SB431542 to block Nodal signaling prior to transplantation and returned to SB431542 containing media following transplantation. SB431542 inhibits Alk4, Alk5 and Alk7 kinase activity, and it has been shown to phenocopy Nodal pathway mutants [19,20].

**Figure 6:**
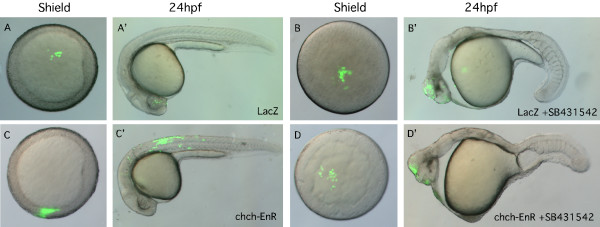
Alk receptor signaling is required for both migration and acquisition of mesodermal character of *chch*-EnR transplanted cells. Transplanted LacZ cells were present in the animal pole at shield stage (A) and were found in anterior neural tissue after 24 hpf (A'). 400 μM SB431542 (Sigma-Aldrich), which inhibits Alk receptors did not alter LacZ donor cell behavior (B-B'). Conversely, *chch*-EnR donor cells moved from the animal hemisphere to the germ ring by shield stage (C) and were observed in mesodermal structures after 24 hpf (C'). SB431542 treatment results in blocking movement of *chch*-EnR cells. The donor cells remain in the animal hemisphere at shield stage (D) and are found in anterior neural structures after 24 hpf (D'). This result suggests that signaling via Alk receptors is required for both migration and acquisition of mesodermal character of *chch*-EnR donor cells. The images in each row are of the same embryo.

As expected, LacZ expressing cells transplanted from the animal hemisphere of a sphere stage (4 hpf) embryo to the animal hemisphere of a wild-type host at a late blastula stage (4.7 hpf) remained in the animal hemisphere (22/22 embryos) and by 1 dpf were observed in anterior neural tissue (Fig. 6A–A' and Table 4). Treatment with SB431542 did not alter the behavior of these cells (Fig. 6B–B'). Conversely, in the same transplant scheme, *chch*-inhibited cells often migrated to the germ ring and became incorporated into mesodermal structures at 1 dpf (Fig. 6C–C' and Table 4). Surprisingly, when Nodal signaling was blocked, *chch*-EnR donor cells remained in the animal hemisphere (32/32 embryos) and were observed in anterior neural structures at 1 dpf (24/32 hosts, Fig. 6D–D' and Table 4). In the remaining 8/32 cases, donor cells were scattered in superficial layers of the yolk (likely epidermis). The behavior of this subset of cells differs from LacZ donors and non-SB431542 treated *chch*-EnR donors and may stem from an incomplete block of Nodal signaling. These findings demonstrate that signaling via Alk receptors is required for both migration to the germ ring and acquisition of mesodermal character of the *chch*-EnR donor cells. Since SB431542 treatment blocked Alk signaling in both donor and host cells, we could not infer which cells required Alk-mediated signaling from these experiments.

**Table 4:** Transplantation of *chch*-EnR cells requires nodal signaling for cell movements and fate changes

			**Location of cells at shield**		**Location of Cells at 24 hpf**				
			
**Donor cells**	**Host embryos**	**n**	**Animal pole**	**Germ ring**	**Neural**	**Epidermis**	**Somites**	**Notochord**	**Yolk (Superficial)**
LacZ	Wild-type	22	22	0	19	3	0	0	0
LacZ	Wild-type + SB431542	25	25	0	25	0	0	0	0
*chch*-EnR	Wild-type	32	19	13	11	1	17	2	0
*chch*-EnR	Wild-type + SB431542	32	32	0	24	0	0	0	8
*MZoep*-LacZ	Wild-type	36	35	1	29	7	0	0	0
*Mzoep-chch*-EnR	Wild-type	48	48	0	41	6	1	0	0
LacZ	Wild-type	26	26	0	23	3	0	0	0
*chch*-EnR/xFsast1-EnR	Wild-type	38	38	0	26	6	0	0	6

To establish whether Nodal signaling is required in the donor or host cells we employed two manipulations to repress Nodal signaling in the donor cells. These experiments utilized MZ*oep *mutants which lack Oep, an EGF-CFC protein that functions as a co-receptor for Nodal signals [3] or dominant-negative xFAST1-EnR mRNA. MZ*oep *embryos are completely non-responsive to Nodal signals. Fast1 (or FoxH1) is a transcription factor that responds to Nodal and FAST1-EnR constructs are effective in blocking Nodal signaling in fish and frogs [21,22].

**Figure 7:**
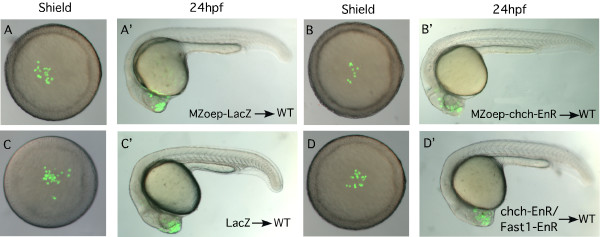
***chch*-compromised cells migrate to the germ ring and become mesoderm in response to Nodal signals**. Transplanted MZ*oep*-LacZ cells to the animal hemisphere of a wild-type host remain within the animal hemisphere at shield stage (A) and are observed in anterior neural structures after 24 hpf (A'). Similar behavior was observed for transplanted MZ*oep*-*chch*-EnR cells in wild-type hosts (B-B'). Similarly, when donor cells express 12.5 pg xFast1-EnR, along with *chch*-EnR, migration to the margin is blocked and donor cells are observed in anterior neural structures after 24 hpf (D-D'). Together, these results demonstrate that the migration of the *chch *inhibited cells to the germ ring and acquisition of mesodermal character depends upon Nodal signaling.

Donor cells were removed at 4 hpf either from the animal pole of an MZ*oep *embryo injected with *chch*-EnR mRNA or from a wild-type donor injected with *chch*-EnR and xFast1-EnR mRNA. First, control or *chch*-inhibited MZ*oep *cells were transplanted to the animal hemisphere of a wild-type host embryo at a late blastula stage (4.7 hpf). LacZ injected MZ*oep *cells behaved similarly to wild-type cells. These donor cells did not leave the animal hemisphere and were observed in anterior neural structures at 1 dpf (Fig. 7A–A'). Similar to the SB431542 experiment, *chch*-EnR-MZ*oep *cells remained in the animal hemisphere (48/48 embryos), and were observed in anterior neural tissues at 24 hpf (Fig. 7B–B' and Table 4) demonstrating that Oep is required for both migration and fate change of the *chch*-EnR donor cells.

We next transplanted cells from donor embryos co-injected with *chch*-EnR and xFast1-EnR to wild-type hosts. Following transplantation of LacZ cells, little movement of donor cells was observed at 6 hpf and donor cells were found in anterior neural structures at 1 dpf (Fig. 7C–C' and Table 4). Similarly, transplanted *chch*-EnR/xFast1-EnR co-injected donor cells (Fig. 7D–D' and Table 4) remained in the animal hemisphere and gave rise to anterior neural tissue (26/38 embryos), epidermis (6/32 embryos) or were on the superficial surface of the yolk (6/32 embryos). This result suggests that xFast1-EnR prevents *chch*-EnR cells from adopting mesodermal fates. However, some *chch*-EnR/xFast1-EnR co-injected donor cells behave differently than the LacZ donors and are found spread across the yolk at 1 dpf. These donor cells likely retain some responses to Nodal since some Nodal signaling events are independent of xFast1 and are instead mediated by Mixer-related molecules [23].

Since *chch*-compromised cells fail to migrate to the germ ring and to assume mesodermal fates if Alk, Oep or Fast1 function is abrogated, we conclude that both migration and fate change depends on Nodal signaling. In the xFast1-EnR and SB431542 experiments, some *chch*-EnR cells underwent aberrant movements but were not later observed in mesodermal structures suggesting that migration and fate change can be uncoupled.

### chch-EnR embryos have enhanced response to Nodal

**Figure 8:**
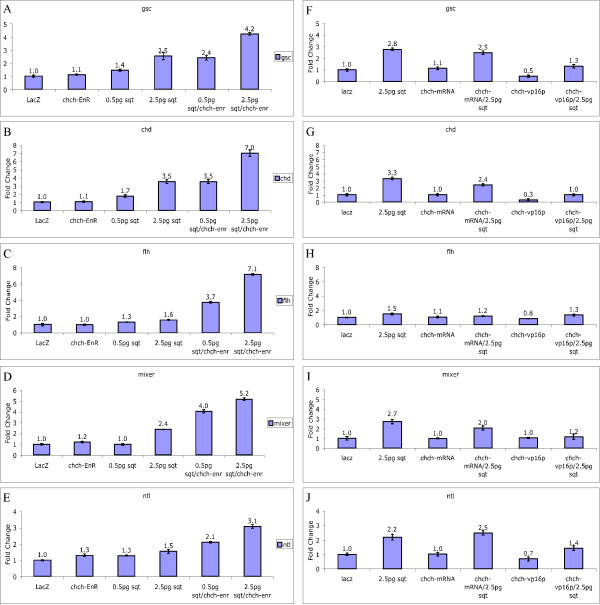
***chch *suppresses the transcriptional response to Nodal signaling**. Embryos were microinjected with either 250 pg *chch*-EnR mRNA, 0.5 pg *sqt *mRNA, 2.5 pg *sqt *mRNA, or co-injected with 250 pg *chch*-EnR mRNA and 0.5 pg or 2.5 pg *sqt *mRNA and were analyzed at shield stage by real-time PCR with the dorsal markers *gsc *(A), *chd *(B), and *flh *(C), the endodermal marker *mixer *(D) and the pan-mesodermal marker *ntl *(E). All of these markers show little change in gene expression in embryos injected with *chch*-EnR mRNA and a dose dependent response to *sqt *mRNA. When both *chch*-EnR and *sqt *mRNA were co-injected, synergistic increases in marker expression were observed. These results demonstrate that *chch *suppresses the response to Nodal signaling. Conversely, microinjection of *chch*-VP-16 mRNA suppresses the transcriptional response to Nodal signaling. Embryos were microinjected with 2.5 pg *sqt*, 500 pg *chch *mRNA, 500 pg *chch*-VP16 mRNA, or co-injected with *sqt *and *chch *mRNA or *chch*-VP16 mRNA. Embryos were collected at shield stage and analyzed by real-time PCR with *gsc *(F), *chd *(G), *flh *(H), *mixer *(I), and *ntl *(J). Co-injection of *chch*-VP16 mRNA along with *sqt *mRNA supresses the transcriptional response to *sqt*.

The above results suggest that the *chch*-EnR cells placed in the animal hemisphere respond to a Nodal signal but wild-type cells do not. Since Nodal ligands are expressed at the margin during late blastula and early gastrula stages, we tested the hypothesis that *chch*-compromised cells have increased responsiveness to low levels of Nodal. Wild-type embryos were microinjected with *chch*-EnR mRNA, 0.5 pg or 2.5 pg of *sqt *mRNA, *chch*-EnR mRNA and *sqt *mRNA or LacZ mRNA. Embryos were collected at shield stage and the levels of five genes whose expression depends on Nodal signaling were measured by real-time PCR. These genes included the dorsal markers, *chd*, *gsc*, *flh*, as well as the pan-mesodermal marker *ntl *and the endodermal marker *mixer *(Fig. 8). All of these markers show no change or modest changes in embryos injected with *chch*-EnR mRNA and a dose-dependent response to *sqt *mRNA (Fig. 8A–E). Synergistic increases were observed when both *chch*-EnR mRNA and *sqt *mRNA were co-injected. For example, microinjection of 2.5 pg of *sqt *mRNA resulted in a 3.5 fold increase in *chd *mRNA levels (Fig. 8A). When *chch*-EnR mRNA was coinjected with 2.5 pg of *sqt*, a greater than 7-fold increase in *chd *mRNA levels were measured.

Overexpression of *chch *mRNA had little effect in suppressing the effects of *sqt *overexpression on the same markers when assayed by real-time PCR at shield stage (Fig. 8F–J). However, we found that overexpression of zebrafish *chch *fused to the VP16 transcriptional activator domain (*chch*-VP16) was able to suppress the effects of Nodal activation (Fig. 8F–J). The endogenous levels of *chd*, *gsc *and *ntl *were also suppressed by microinjection of *chch*-VP16 mRNA. Levels of *mixer *and *flh *were unaltered by microinjection of either *chch *mRNA or *chch*-VP16 mRNA. Together results demonstrate that *chch *functions to suppress the response to Nodal signaling.

## Discussion

We characterized the function of the zinc finger transcription factor Churchill in zebrafish. Our data showed that *chch *is required to repress expression of markers of non-axial mesoderm, while both neural and presumptive epidermal markers are diminished in *chch*-compromised embryos. In transplant assays, cells with compromised *chch *function undergo atypical cell movements when placed in the animal hemisphere and acquire fates inappropriate for their position in the early gastrula. Unlike control cells, these donor cells often migrate to the margin and are later observed in mesodermal derivatives. The movement and corresponding fate change of the transplanted *chch *compromised cells can be suppressed by blocking Nodal signaling in the donor cells. Finally, we demonstrate that *chch *suppresses the transcriptional response to Nodal signaling.

### chch limits expression of mesodermal markers

In *Xenopus*, *chch *overexpression is sufficient to repress *Xbra *expression [15]. Our data demonstrate that when *chch *function is repressed, mesodermal gene expression is expanded during gastrulation (Fig. 2) and the expansion corresponds to a decrease in the levels of ectodermal markers (Fig. 3). The effects on ectoderm are relatively modest during gastrulation but at later stages anterior neural defects were observed.

In contrast to *Xenopus*, we did not detect repression of *ntl *(*brachyur*y) when *chch *is overexpressed (Fig. 2I–J). Since the concentration of *chch *mRNA used in the overexpression experiments was sufficient to rescue both the morpholino and dominant-negative phenotypes, these levels are likely physiologically relevant. Overexpression of higher doses of *chch *mRNA failed to alter early *ntl *expression (data not shown). However, we do observe repression of *ntl *following microinjection of *chch*-VP16 mRNA (Fig. 8). The different effects of *chch *overexpression on *brachyury *expression may reflect experimental differences or divergence in regulation of *brachyury *between fish and frogs. One possibility is that an essential *chch *or Sip1 cofactor is widely expressed in *Xenopus *but has restricted expression in zebrafish.

### chch regulates cell movement

We show that cells with compromised *chch *function undergo inappropriate cell movements. When placed into a wild-type embryo at a late blastula stage, these cells often leave the epiblast, migrate to the germ ring and become mesoderm (Fig. 5). This result is consistent with the *chch *study in the chick, where *chch *was shown to be required to repress ingression of epiblast cells into the primitive streak [15]. These data suggest an evolutionarily conserved function for *chch *in the regulation of cell movement during gastrulation.

A key question is whether the *chch *compromised cells that leave the epiblast and enter the germ ring, become mesoderm because *chch *regulates migration and they are subsequently exposed to mesoderm-inducing signals in the germ ring or whether they migrate to the germ ring because they have already established mesodermal identity; is movement directing fate or is fate controlling movement?

The transplant experiments in which we manipulated the responsiveness of the donor cells to Nodal signaling revealed that *chch*-EnR cells do not autonomously become mesoderm but require activation of the Nodal pathway to migrate to the germ ring and adopt mesodermal fates. When Nodal signaling is blocked, *chch*-compromised cells fail to migrate to the germ ring and do not assume mesodermal fates (Fig 6, 7). Analysis of zebrafish Nodal mutants has shown that Nodal signaling is required for internalization of mesodermal precursors [24]. Our results suggest that Nodal may also play a role in cell movement toward the margin prior to internalization. The Nodal family member responsible for this activity has not been established, although Sqt is a strong candidate because it is expressed at these stages and has been shown to act as a long-range signal [25]. Alternatively, the movement of *chch*-compromised donor cells may be influenced by another Nodal family member, perhaps Vg1 which is also expressed early and requires Oep function [26,27].

It is important to note that not all cells that underwent unusual movements ended up in the mesoderm. This implies that movement can be uncoupled from acquisition of mesodermal character. Since not all of the donor cells showed the same movements, the differences may have resulted in variations in the initial positions of cells or subtle differences in *chch *activity in the donor cells. In addition to an enhanced responsiveness to Nodal signals, *chch*-compromised cells might also be sensitive to other TGF-β signals including BMPs, which could account for alterations in cell behavior. More detailed analysis of the behavior of *chch*-deficient cells will be necessary to determine additional roles for *chch *in regulating cell movements during gastrulation.

### chch represses the transcriptional response to Nodal

We found that the transcriptional response to the Nodal ligand *sqt *is enhanced in embryos expressing *chch*-EnR mRNA. The mRNA levels of five Nodal target genes were synergistically increased when *sqt *and *chch*-EnR mRNA where co-injected (Figure 8). Three of these targets, *chd*, *fh *and *gsc *are expressed in the dorsal mesoderm. The fourth, *mixer*, is an endodermal marker. However, in contrast to pan-mesodermal markers like *ntl *(Fig. 2 and 8) and *spt *(Fig. 2), the transcript levels of these markers were largely unaltered by *chch*-EnR mRNA or *chch*-ATGMO microinjection. This suggests that endogenous *chch *does not play a role in suppressing the high levels of Nodal signaling that are required for specification of dorsal mesoderm and endoderm but represses the response to lower Nodal levels.

Our data suggests that the increased expression of mesodermal markers observed when *chch *is repressed results from an enhanced response to Nodal. Microinjection of morpholinos directed against the extracellular Nodal antagonist, Lefty, also result in expansion of mesodermal markers [5,28,29]. The *chch *target Sip1 likely mediates the effect of *chch *on Nodal signaling. Sip1 has not been previously characterized in zebrafish, although we established that zebrafish Sip1 is regulated by *chch *(Fig. 2K–N, Q). Sip1 represses TGF-β signaling by binding to activated forms of Smad1/5 and Smad2/3 [30,31] and is a direct repressor of *Xbra *[32]. The decrease in Sip1 expression following *chch *inhibition could result in a failure to check Nodal signaling, which results in mesoderm expansion. However, Sip1 morpholinos do not alter Xbra expression in *Xenopus *[33]. The increase in mesodermal gene expression we observe in *chch*-compromised embryos (Fig. 2) is consistent with an enhanced sensitivity to TGF-β signals. In addition to suppressing *Xbra *and TGF-β signaling, Sip1 is also a direct repressor of *E-Cadherin *[34]. While migration of *chch*-compromised cells to the germ ring depends on Nodal signaling, alterations in *E-cadherin *levels may also influence the unusual movements of these cells.

## Conclusion

Our study of the zinc finger transcription regulator, *chch*, is the first analysis of the function of this gene in zebrafish. We have discovered roles for *chch *in regulating cell movements within the gastrula that are consistent with the initial data on *chch *in the chick and *Xenopus*. Significantly, we have identified several novel functions for *chch*. Our data suggests a broader role for *chch *then was previously demonstrated and provides key insight into the mechanism of action of *chch*. From this analysis we conclude: (1) that *chch *is required to limit mesodermal gene expression; (2) *chch *inhibits Nodal-dependant movement of presumptive ectodermal cells (3) *chch *represses the transcriptional response to Nodal signaling. These findings provide a basis to begin to elucidate the dynamic roles for *chch *in regulating cell movement and fate during early development.

## Methods

### Constructs and morpholinos

The Churchill coding sequence was cloned into the StuI site of the pCS2 plasmid, pCS2-EnR (*chch*-EnR) or pCS2-VP16 (*chch*-VP16). Sense mRNA was made using the mMESSAGE mMACHINE RNA synthesis kit (Ambion). Morpholinos were synthesized by GeneTools (Philomath, OR): *chch*-ATGMO-5'-GCTTCTGGACACAACCGGTACACAT

### RNA in situ hybridization and photography

RNA *in situ *hybridization, probes and photography techniques were previously described [7].

### Real-time PCR

PCR and primers were previously described [7], except *chch*: F-5'-TGTGTCCAGAAGCAATATCC, R-5'-TCCTCCTCATCTTCATTCAC; Sip1: F-5'-CACTCAGCTGGAGAGACATA, R-5'-TGCTCCTTTAGATGGTGTTT; Mixer F-5'-CAGAATCGAGAATTCAGGTC, R-5'-TGTGGTAAACTGGTGCATAA.

### Cell transplants

Embryos were microinjected with either LacZ mRNA for a control *chch*-ATGMO, or *chch*-EnR mRNA for the experimentals along with 5 mg/ml Fluorescein dextran (Molecular Probes). For the isochronic transplants, cells from sphere stage embryos were transplanted to a sphere stage host embryo. For the heterochronic transplants, sphere stage cells were transplanted to the animal hemisphere of 30% epiboly embryos. Each transplant consisted of 5–15 cells. The location of the transplanted cells was scored and documented immediately after the transplantation and again at shield stage (early gastrulation). Embryos were sorted based upon the location of the transplanted cells relative to the shield (in the shield, lateral or ventral to the shield, or in the ectoderm), and photographed. The location of the cells was observed again after 24 hrs and documented.

## Authors' contributions

EL carried out the transplant and real-time PCR experiments, LM carried out the whole mount RNA in situ hybridizations and imaging. HS conceived of the study, and participated in its design and coordination. All authors read and approved the final manuscript.
